# An initial genomic blueprint of the healthy human oesophageal microbiome

**DOI:** 10.1099/acmi.0.000558.v3

**Published:** 2023-06-26

**Authors:** Rachel Gilroy, Mina E. Adam, Bhaskar Kumar, Mark J. Pallen

**Affiliations:** ^1^​ Quadram Institute Bioscience, Norwich Research Park, Norwich, UK; ^2^​ Norfolk & Norwich University Hospitals NHS Foundation Trust, Norwich, UK; ^3^​ School of Veterinary Medicine, University of Surrey, Guildford, Surrey, UK; ^4^​ University of East Anglia, Norwich Research Park, Norwich, UK

**Keywords:** metagenome-assembled genome, metagenomics, microbiome, oesophageal microbiome, oesophagus

## Abstract

**Background.:**

The oesophageal microbiome is thought to contribute to the pathogenesis of oesophageal cancer. However, investigations using culture and molecular barcodes have provided only a low-resolution view of this important microbial community. We therefore explored the potential of culturomics and metagenomic binning to generate a catalogue of reference genomes from the healthy human oesophageal microbiome, alongside a comparison set from saliva.

**Results.:**

Twenty-two distinct colonial morphotypes from healthy oesophageal samples were genome-sequenced. These fell into twelve species clusters, eleven of which represented previously defined species. Two isolates belonged to a novel species, which we have named *Rothia gullae*. We performed metagenomic binning of reads generated from UK samples from this study alongside reads generated from Australian samples in a recent study. Metagenomic binning generated 136 medium or high-quality metagenome-assembled genomes (MAGs). MAGs were assigned to 56 species clusters, eight representing novel *Candidatus* species*,* which we have named *Ca*. Granulicatella gullae, *Ca*. Streptococcus gullae, *Ca*. Nanosynbacter quadramensis, *Ca*. Nanosynbacter gullae, *Ca*. Nanosynbacter colneyensis, *Ca*. Nanosynbacter norwichensis, *Ca*. Nanosynococcus oralis and *Ca*. Haemophilus gullae. Five of these novel species belong to the recently described phylum *Patescibacteria *. Although members of the *Patescibacteria* are known to inhabit the oral cavity, this is the first report of their presence in the oesophagus. Eighteen of the metagenomic species were, until recently, identified only by hard-to-remember alphanumeric placeholder designations. Here we illustrate the utility of a set of recently published arbitrary Latinate species names in providing user-friendly taxonomic labels for microbiome analyses.

Our non-redundant species catalogue contained 63 species derived from cultured isolates or MAGs. Mapping revealed that these species account for around half of the sequences in the oesophageal and saliva metagenomes. Although no species was present in all oesophageal samples, 60 species occurred in at least one oesophageal metagenome from either study, with 50 identified in both cohorts.

**Conclusions.:**

Recovery of genomes and discovery of new species represents an important step forward in our understanding of the oesophageal microbiome. The genes and genomes that we have released into the public domain will provide a base line for future comparative, mechanistic and intervention studies.

## Full-Text

## Data availability

The datasets supporting the conclusions of this article are available in the NCBI SRA database under BioProject ID PRJNA838635 and BioProject ID PRJEB25422. We have made further information available in the FigShare database https://doi.org/10.6084/m9.figshare.19786234 [1].

## Background

The human oesophagus is a fibromuscular tube that connects the pharynx to the stomach. Oesophageal cancer is the sixth leading cause of death from cancer, causing over half a million deaths per year globally [2]. The oesophagus is home to a complex microbial community – the oesophageal microbiome – that potentially contributes to the pathogenesis of oesophageal cancer [3]. However, investigations using culture and molecular barcodes have, so far, provided only a limited, low-resolution view of taxonomic and functional diversity within this community [4]. This means that important biological roles remain undiscovered, with limited opportunities for hypothesis generation and testing. It also remains unclear how far the oesophageal microbiome is distinct from that of the oral cavity, rather than simply representing the salivary microbiome in transit through the oesophagus [5].

Culturomics – combining high-throughput culture under a range of laboratory conditions with whole-genome sequencing – provides an attractive route to generation of high-quality bacterial genomes from complex microbial communities [6]. However, as many microbial species evade cultivation, a comprehensive microbial census of the oesophagus is likely to require additional culture-independent approaches, such as shotgun metagenomics [4].

Deshpande and colleagues have recently applied shotgun metagenomic sequencing to oesophageal samples, followed by reference-based phylogenetic profiling [7]. However, such phylogenetic profiling relies on a reference database and so can only report previously known organisms and can never uncover ‘unknown unknowns’, i.e. inhabitants of the oesophagus not seen elsewhere. In addition, reference-based profiling provides limited insights into the functional diversity or population structure of microbial species and is prone to artefacts [8].

Studies on the lower gut and skin have shown that generation of metagenome-assembled genomes (MAGs) from metagenomic datasets provides a powerful reference-free approach to the characterisation of taxonomic and functional diversity within complex microbial communities [9, 10]. With that in mind, here we explore the methodological potential of culturomics combined with the creation of MAGs to generate a preliminary catalogue of reference genomes from the healthy human oesophageal microbiome, alongside a comparison set of MAGs from saliva. We were surprised to find remarkable novel microbial diversity in this commonplace setting.

## Methods

### Sample collection

**Fig. 1.**
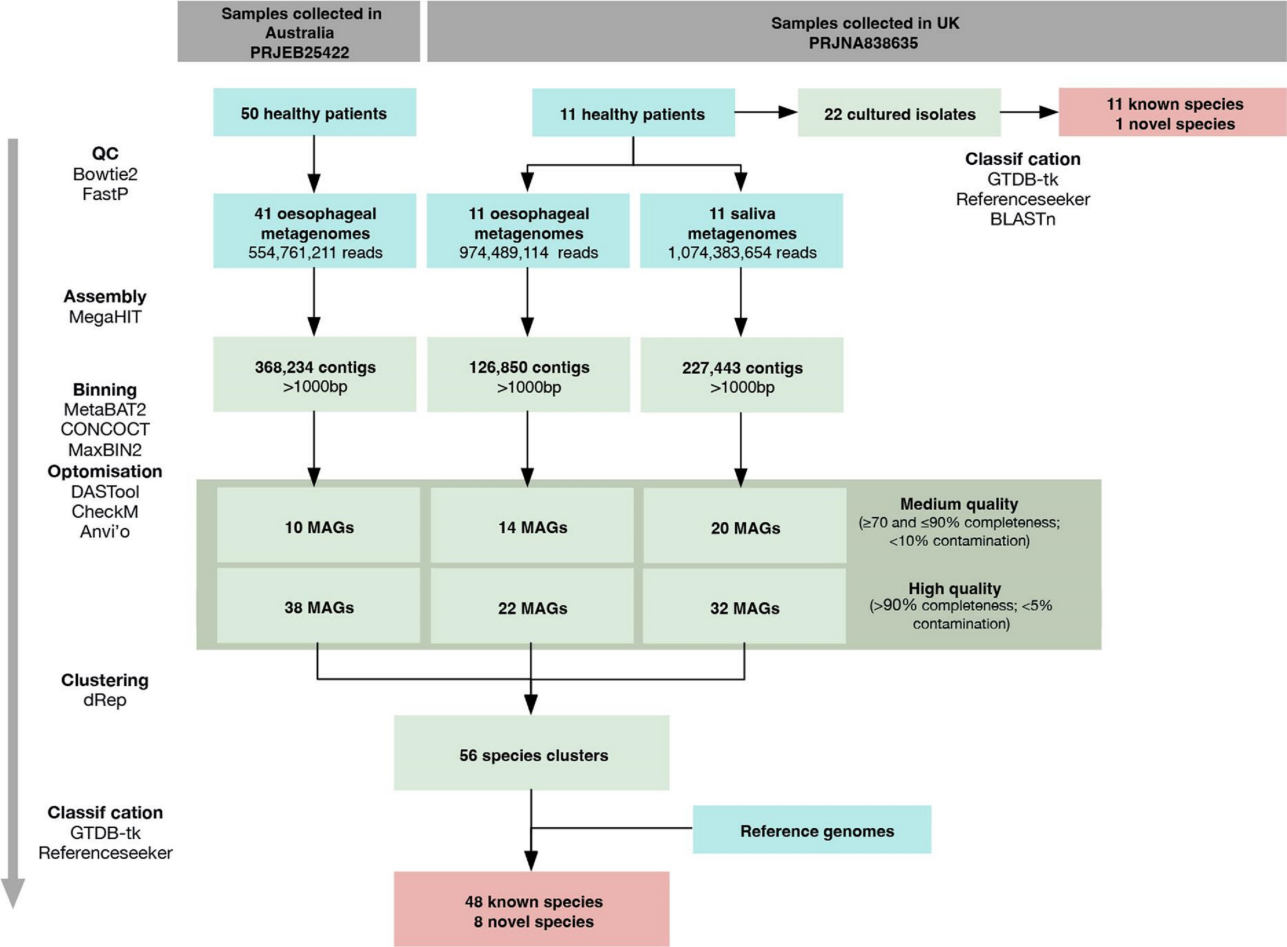
Analytical workflow. The core bioinformatic flow diagram.

The workflow for this study is outlined in Fig. 1. Eleven patients were prospectively recruited while undergoing upper gastrointestinal endoscopy at the Norfolk and Norwich University Hospital, Norwich, UK. All participants provided informed written consent and the study was conducted with ethical approval from the University of East Anglia’s Faculty of Medicine and Health Sciences Research Ethics Subcommittee (Application ID: ETH2122-0626). Study inclusion was dependent on participants presenting with a normal oesophagus with no sign of pathology at endoscopy. Exclusions included previous upper gastrointestinal surgery or use of antibiotics or non-steroidal anti-inflammatory drugs in the 2 months prior to the procedure. Use of mouthwash, eating and drinking were not permitted in the 4 h before endoscopy. The participants included five females and six males, ranging from 20 to 83 years old (Table S1, available in the online version of this article). A single saliva sample and three oesophageal brushings were collected per subject. Mucosal brushings of the oesophagus collected in this way have shown higher microbial DNA and reduced human DNA contamination compared to oesophageal biopsies [11]. Two oesophageal brushes were pooled for metagenomic sequencing while the remaining brush was used for bacterial culture.

### Bacterial culture

Sample processing occurred within 4 h of collection, with oesophageal brushes added to a sterile 2 ml polypropylene tube containing 1.5 ml phosphate-buffered saline. Samples were gently vortexed for 1 min, before 200 µl extracts were spread on to two types of agar (Brain Heart Infusion [BHI], Sigma-Aldrich; Colombia Blood Agar [CBA], Sigma-Aldrich; Table S2). Cultures were incubated at 37 °C for 72 h. Colonies were picked every 24 h, selecting colonial morphotypes distinctive in colour, shape and size. Cultures from colony picks were re-streaked on a fresh agar plate containing the growth medium from which they were first isolated to confirm purity. Individual colonies were inoculated into 2 ml of broth (mirroring their source culture medium) before incubation at 37 °C for 24 h. All isolates were archived at −80 °C in 20 % glycerol.

### Cultured genome sequencing and bioinformatic analysis

DNA extraction was performed on 200 µl of overnight bacterial culture using the Maxwell RSC cultured cell kit (Promega Corporation, Madison, WI) according to manufacturer’s instructions. DNA was quantified using a Qubit fluorometer (Invitrogen, Carlsbad, CA, USA) high-sensitivity assay, before dilution to the required concentration using RNase-free water and purification on AMPure XP beads (Beckman Coulter, Brea, CA, USE). Twenty-two bacterial isolates produced high-quality DNA and were selected for whole-genome sequencing. Sequencing library preparation and whole genome sequencing using the Illumina NextSeq were performed as described previously [12].

Paired-end reads were quality assessed and trimmed using FastP v0.23.2 (fastp, RRID:SCR_016962) [13], before assembly of high-quality reads using SPAdes v3.15.3 (SPAdes, RRID:SCR_000131) [14]. Only scaffolds >1000 bp were included in downstream analysis. CheckM (CheckM, RRID:SCR_016646) v1.1.10 [15] was used to attain completeness and contamination scores for each assembled genome, with only those genomes according to criteria described previously by Gilroy *et al*. [12] confirmed as passing quality control thresholds. Genomes were clustered according to Average Nucleotide Identity (ANI) at 95 % according to commonly used pre-defined species level thresholds [16]. Taxonomic assignment of recovered species was performed according to the Genome Taxonomy Database Toolkit (GTDB-Tk, RRID:SCR_019136) v2.0.0 on GTDB Release 207 v2 [17] and ReferenceSeeker v1.8.0 (NCBI RefSeq release 201) [18] (BioRxiv, 863621). Barrnap v0.9 (Barrnap, RRID:SCR_015995) was applied to all genomes passing quality filters for extraction of full-length 16S rRNA gene sequences before comparison against NCBI bacterial and archaeal 16S rRNA references using the web-based blastn tool (blastn, RRID:SCR_001598) [19]. For isolates showing no definitive known representative, FastANI v1.33 [20] was applied for ANI comparison against all closely related species retrieved from NCBI.

### Metagenomic DNA enrichment, extraction and sequencing

Microbial DNA enrichment and host DNA depletion was performed on pooled oesophageal brushings using the MolYsis Basic5 kit (Molzym, Bremen, Germany) according to the manufacturer’s instructions, with the resulting cell pellet stored at −20 °C until DNA extraction. Saliva samples were always collected prior to the collection of oesophageal brushings and stored at 4 °C in 1 : 1 DNA/RNA shield Solution (Zymo Research) for 24–48 h before DNA extraction. DNA was extracted from saliva and oesophageal brushings using the QIAmp DNA Mini Kit according to manufacturer’s instruction (Qiagen, Hilden, Germany), with DNA fractions eluted in 50 µl of dH_2_O and stored at −20 °C. All oesophageal brush samples were processed within 1–3 h of collection.

DNA quantification was performed using a Qubit 3.0 fluorometer (Invitrogen, CA) and double-stranded DNA (dsDNA) HS assay kit. Pooled Illumina sequencing libraries were constructed according to methods previously described by Ravi and colleagues [21]. Paired-end metagenomic sequencing was performed on the Illumina Novaseq 6000 platform yielding 2×250 bp paired-end sequencing reads.

### Mapping to the human genome and read-based analysis

Drawing on NCBI BioProject PRJEB25422, associated with the study by Deshpande and colleagues [7], we incorporated a further 50 metagenomes sourced from oesophageal bush samples of healthy Australian patients to our dataset. Bioinformatics analysis was performed on the Cloud Infrastructure for Microbial Bioinformatics [22]. Metagenomic reads were trimmed, and quality controlled using FastP (fastp, RRID:SCR_016962) configured to a minimum phred score of 20 and minimum length of 50 bp [13]. Trimmed reads were mapped to the human genome assembly GRCh38.p13 (GCA _000001405.28) using Bowtie2 v2.3.5.1 [23], with all host-associated reads removed from downstream analysis by SAMtools v1.7 (SAMTOOLS, RRID:SCR_002105). Host-depleted metagenomic sequences from our 11 patients can be accessed from BioProject PRJNA838635. Nine samples from BioProject PRJEB25422 had a host-depleted read count of <500000 and were removed from further analysis creating a final sample catalogue of 52 oesophageal metagenomes and 11 saliva metagenomes (Table S3).

### Metagenomic assembly, binning and refinement

Individual assembly was performed on all metagenomes from the combined dataset using MegaHIT v1.2.9 (MEGAHIT, RRID:SCR_018551) before quality assessment of the resulting contiguous sequences using Anvi’o v7.1 [24]. Contigs <1000 bp in length were removed from all assemblies. Assembly abundance profiles were generated by mapping filtered reads against their respective assemblies using Bowtie2 [23], processing the resulting SAM file to create a sorted and indexed BAM file using SAMtools [25]. Single sample binning was performed using three automated binning tools MaxBin2 v2.2.7 [26], MetaBAT2 v2.15 [27] and CONCOCT v1.1.0 [28] according to contig coverage depth, before optimisation of the resulting bin catalogue with DAS Tool v1.1.4 [29]. The resulting bins recovered from our 63 metagenomic samples were refined according to GC content, coverage and single copy core gene (SCG) content using Anvi’o ‘anvi-profile’ and ‘anvi-refine’ workflows (Anvi'o, RRID:SCR_021802) as previously described [24]. CheckM (CheckM, RRID:SCR_016646) [15] was used for quality assessment of all bins using the lineage_wf function. Bins showing >50  % completion and <10  % contamination were assessed for quality score (defined as estimated genome completeness score minus five times estimated contamination score), a commonly used standard for defining acceptable bin quality [30]. Bins with <70  % completion and/or a quality score of <50 were categorised as low-quality MAGs; those with >70  % completion, <10  % contamination and quality score >50 were categorised as medium-quality MAGs and those with >90  % completion, <5  % contamination and quality score >50 were classified as high-quality MAGs (Table S4). To estimate the completeness and contamination of suspected members of Candidate Phyla Radiation (CPR), we used 43 CPR specific markers [31] within CheckM retaining the quality thresholds described above for larger genomes.

Medium- and high-quality MAGs were de-replicated at 95 % ANI with a default aligned fraction of>10 % using dRep v2.0 [16], to create a non-redundant species catalogue. GTDB-Tk [17] and ReferenceSeeker [18] were used to perform taxonomic assignment of recovered MAGs compared to the ‘Release 207 v2’ and NCBI ‘RefSeq release 201’ databases, respectively (Table S5). We used a modified version of the GTDB taxonomy file recently described by Pallen *et al.* [32] that included well-formed Latinate *Candidatus* names rather than the default alphanumeric designations. Species recovered from both the oesophagus and the saliva were compared for similarity using FastANI [20] and viewed using the R package ggPlot2 [33].

### Phylogenetic placement of recovered species

All novel species clusters were confirmed as monophyletic, drawing on all publicly available genomes from the genus to which they had been assigned by GTDB (with genomes retrieved by NCBI). Proteomes were predicted using Prodigal v2.6.1 (Prodigal, RRID:SCR_011936) [34] before comparison against 400 universal marker proteins using PhyloPhlAn v3.0.58 (PhyloPhlAn, RRID:SCR_013082) [35] in accordance with diamond v0.9.34 (DIAMOND, RRID:SCR_016071). Multiple sequence alignment and subsequent refinement was performed using MAFFT v7.271 (MAFFT, RRID:SCR_011811) [36] and trimAl v1.4 (trimAl RRID:SCR_017334) [37]. Where whole genome alignments were required, these were performed using progressiveMauve [38], with non-conserved regions >20 kbp queried using blastn [19]. Abundance of these non-conserved sequences was determined by mapping host-depleted metagenomic reads using Bowtie2 (Bowtie 2, RRID:SCR_016368) [23] before creation of a coverage profile using CheckM [15].

When no cultured isolates were available, the representative genomes selected for inclusion in the final non-redundant species catalogue were chosen based on quality score. A phylogeny for our final de-replicated species catalogue was constructed by aligning and concatenating a set of sixteen ribosomal protein sequences (ribosomal proteins L1, L2, L3, L4, L5, L6, L14, L16, L18, L22, L24, S3, S8, S10, S17 and S19) [39]. Ribosomal sequences were extracted using anvi’o [24] before alignment using muscle v3.8.1551 (muscle, RRID:SCR_011812) [40] and refinement using trimAl v1.4 [37]. A maximum-likelihood tree was constructed using FastTree v2.1 (FastTree, RRID:SCR_015501) [41]. All trees were visualised and manually annotated using iTol v5.7 (iTOL, RRID:SCR_018174) [42] (Table S1).

### Relative abundance estimation and functional annotation of MAGs

To determine mean coverage and relative abundance our non-redundant species catalogue within saliva and oesophageal brush metagenomes, host-depleted metagenomic reads from each sample were mapped back to our concatenated non-redundant species catalogue using Bowtie2 [23]. Absence/presence of a species within any given metagenome was determined at 1X mean genome coverage (proportion of nucleotides in a genome covered by at least one read) over at least 25 % of the genome length. Relative abundance of any given species was estimated according to previously described methods [43]. Briefly, total reads mapping to a single species was divided by the total number of reads in that sample, before further dividing by species length in Mbp. All reads not mapping to our non-redundant MAG catalogue were assigned as an ‘unknown’ bin of assigned length 2Mbp. These abundances were then summed to obtain a sample specific normalising factor by which each previously calculated abundance could be divided to produce a normalised relative abundance value (Table S6). All statistical analysis of the resulting relative abundance table was performed in R using the following packages Vegan [44], Phyloseq [45], ggPlot2 [33]. Bray-Curtis dissimilarity and nonmetric multidimensional scaling (NMDS) was performed on normalised relative abundances, with the significant of association assessed using analysis of similarities (ANOSIM).

## Results

### Genomes from cultured isolates

Thirty-eight colony picks were propagated from the UK oesophageal samples. Sixteen isolates were excluded from further analysis on the grounds of redundancy in colonial morphology, leaving 22 colonial morphotypes isolated, processed and genome-sequenced (Table S2). We were unable to culture any colonies from the oesophageal sample of one patient. Algorithmic clustering identified twelve species clusters at 95 % ANI. Eleven of these were assigned by the GTDB-Tk into previously defined species belonging to four genera. While all these species are known to inhabit the human oral cavity, analysis of the isolation sources of NCBI BioSamples suggests that most of our isolate genomes represent the first genome from the species recovered from the oesophagus (Table 1).

**Table 1.** Bacterial species identified within the microbiome of the healthy human oesophagus and saliva. Only the 54 species assigned to known bacterial species are displayed. Alphanumeric designations from GTDB are listed alongside recently published *Candidatus* names [32]

Species	GTDB alphanumeric placeholder	Type	Source	Subculture	Cultured type strain	Associated with human	Associated with oesophagus	Publication
* Actinomyces graevenitzii *		MAG	Both	na	Yes	Yes	Yes	[49]
*Ca.* Alloprevotella rovamia	Alloprevotella sp000318095	MAG	Oesophagus	na	Yes	Yes	No	
*Ca.* Alloprevotella detaria	Alloprevotella sp015257125	MAG	Oesophagus	na	No	Yes	No	
*Ca.* Alloprevotella dicaposa	Alloprevotella sp015259235	MAG	Oesophagus	na	No	Yes	No	
*Ca.* Alloprevotella abuposa	Alloprevotella sp905369775	MAG	Both	na	No	Yes	No	
*Ca.* Alloprevotella bolacana	Alloprevotella sp905371275	MAG	Oesophagus	na	No	Yes	No	
* Alloprevotella tannerae *		MAG	Saliva	na	Yes	Yes	No	
*Anaeroglobus micronuciformis*		MAG	Saliva	na	Yes	Yes	No	
*Ca.* Butyrivibrio umebia	Butyrivibrio sp015258065	MAG	Saliva	na	No	Yes	No	
*Ca.* Centipeda aniraria	Centipeda sp015265235	MAG	Saliva	na	No	Yes	No	
* Haemophilus seminalis *		MAG	Oesophagus	na	Yes	Yes	No	
*Haemophilus_A parahaemolyticus*		MAG	Oesophagus	na	Yes	Yes	No	
*Haemophilus_D parainfluenzae_K*		MAG	Both	na	Yes	Yes	No	
*Haemophilus_D parainfluenzae_L*		MAG	Saliva	na	Yes	Yes	No	
*Ca.* Clofiposa ofocaria	HOT-345 sp013333295	MAG	Oesophagus	na	No	Yes	No	
* Lachnoanaerobaculum orale *		MAG	Saliva	na	Yes	Yes	No	
* Lancefieldella rimae *		MAG	Oesophagus	na	Yes	Yes	No	
*Ca.* Lancefieldella ubevana	Lancefieldella sp000564995	MAG	Both	na	Yes	Yes	No	
* Limosilactobacillus fermentum *		MAG	Oesophagus	na	Yes	Yes	Yes	[50]
* Neisseria bacilliformis *		MAG	Saliva	na	Yes	Yes	No	
* Neisseria elongata *		Culture	Oesophagus	S181	Yes	Yes	No	
* Neisseria perflava *		Culture	Oesophagus	S144	Yes	Yes	No	
*Ca.* Neisseria efetella	Neisseria sp000186165	MAG	Oesophagus	na	Yes	Yes	No	
*Neisseria subflava_C*		Culture, MAG	Both	S182, S185	Yes	Yes	No	
*Ca.* Pauljensenia ufinia	Pauljensenia sp000278725	MAG	Saliva	na	Yes	Yes	No	
*Ca.* Pauljensenia itixia	Pauljensenia sp000411415	MAG	Saliva	na	Yes	Yes	No	
*Ca.* Pauljensenia epharella	Pauljensenia sp018382595	MAG	Both	na	No	Yes	No	
*Ca.* Pauljensenia gupalia	Pauljensenia sp902373545	MAG	Oesophagus	na	No	Yes	No	
* Porphyromonas endodontalis *		MAG	Both	na	Yes	Yes	Yes	[49]
* Porphyromonas pasteri *		MAG	Both	na	Yes	Yes	No	
* Prevotella histicola *		MAG	Both	na	Yes	Yes	Yes	[51]
* Prevotella intermedia *		MAG	Saliva	na	Yes	Yes	No	
* Prevotella jejuni *		MAG	Both	na	Yes	Yes	No	
* Prevotella melaninogenica *		MAG	Both	na	Yes	Yes	Yes	[49]
* Prevotella nanceiensis *		MAG	Oesophagus	na	Yes	Yes	No	
* Prevotella pallens *		MAG	Oesophagus	na	Yes	Yes	Yes	[49]
* Prevotella salivae *		MAG	Oesophagus	na	Yes	Yes	No	
*Ca.* Prevotella quepia	Prevotella sp000257925	MAG	Oesophagus	na	Yes	Yes	No	
* Rothia dentocariosa *		Culture,MAG	Both	S149	Yes	Yes	No	
* Rothia mucilaginosa *		Culture,MAG	Both	S151	Yes	Yes	Yes	[49]
*Rothia mucilaginosa_A*		Culture,MAG	Both	S145, S153	Yes	Yes	No	
*Ca.* Rothia ivenaria	Rothia sp001808955	Culture,MAG	Both	S183	Yes	Yes	No	
* Simonsiella muelleri *		MAG	Saliva	na	Yes	Yes	No	
* Staphylococcus aureus *		Culture	Oesophagus	S178, S186	Yes	Yes	Yes	[52]
*Stomatobaculum longum_A*		MAG	Saliva	na	No	Yes	No	
* Streptococcus mitis *		MAG	Oesophagus	na	Yes	Yes	Yes	[49]
*Streptococcus mitis_AP*		MAG	Oesophagus	na	Yes	Yes	No	
*Streptococcus mitis_BM*		MAG	Oesophagus	na	Yes	Yes	No	
* Streptococcus salivarius *		Culture	Oesophagus	S175, S180, S143, S173, S172	Yes	Yes	Yes	[52]
*Ca.* Streptococcus ucevana	Streptococcus sp001556435	Culture	Oesophagus	S152	Yes	Yes	No	
* Streptococcus vestibularis *		Culture	Oesophagus	S184, S146, S170	Yes	Yes	Yes	[53]
*Ca.* Tannerella ofiposa	Tannerella sp003033925	MAG	Oesophagus	na	Yes	Yes	No	
*Veillonella parvula_A*		MAG	Oesophagus	na	Yes	Yes	No	
*Ca.* Veillonella ediparia	Veillonella sp900550455	MAG	Saliva	na	No	Yes	No	

**Fig. 2.**
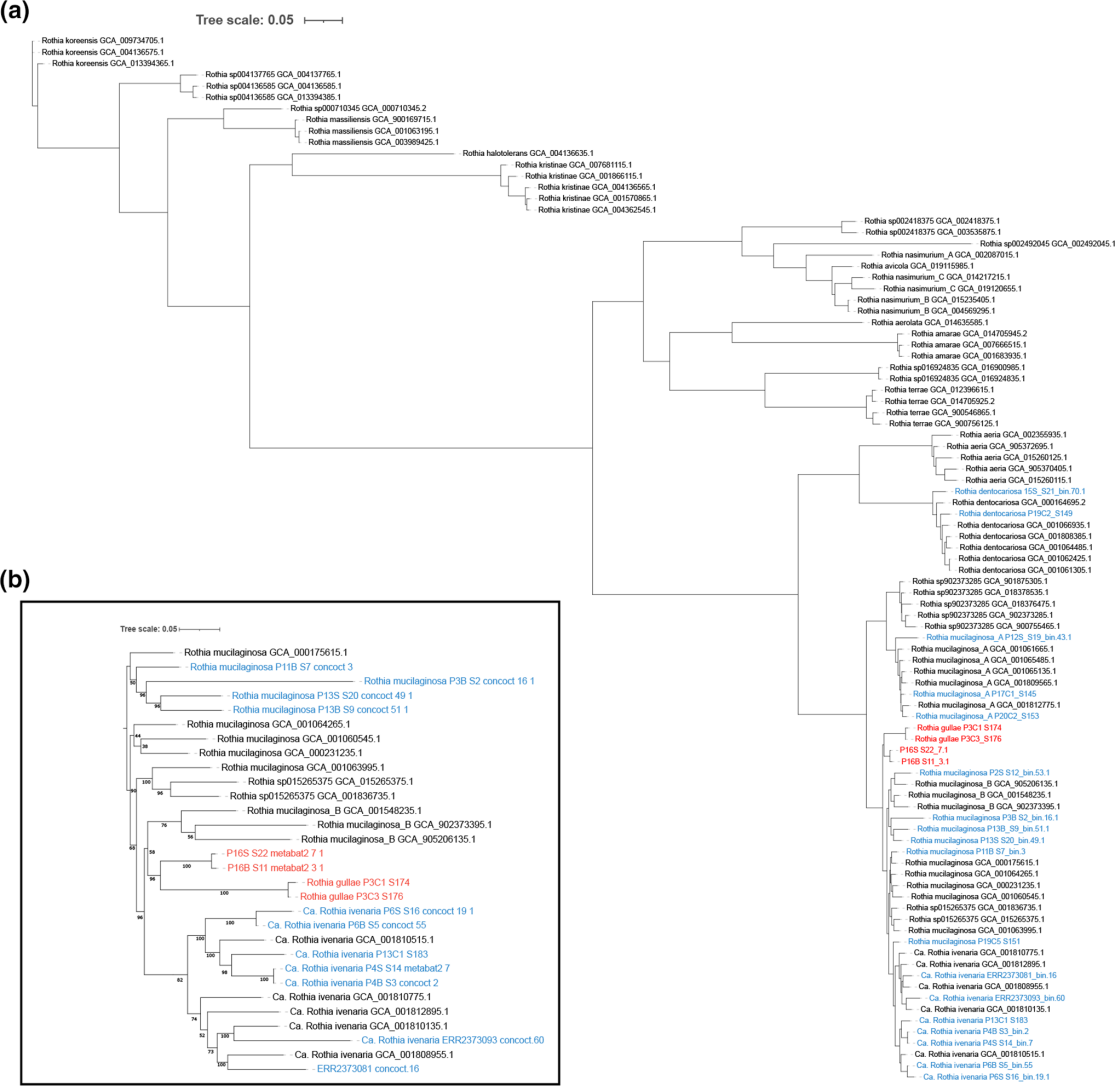
Phylogenetic tree showing the relationships between Rothia species recovered from the healthy human oesophagus and saliva. Trees were constructed using PhyloPhlAN 3.0.58 against 400 marker genes using MAFFT for sequence alignment. (**a**) Tree was reconstructed using FastTree and RAxML. Five reference genomes from all Rothia species listed in GTDB release 207v2 are shown, always inclusive of GTDB species representatives. (**b**) Bootstrapped maximum likelihood tree was reconstructed using mega 11 using the Tamura-Nei model inferring from 100 replicates. Reference strains are listed in black and strains recovered as part of this study in blue and red. Strains highlighted in red are those forming a distinct monophyletic clade indicating novelty. Final trees were visualised and annotated using the online iTOL v5.7 tool.

Two isolates from a single patient were assigned to a species cluster that is closely related to *Rothia mucilaginosa* but sits outside the 95 % ANI radius for the species (Table S7). Phylogenetic analysis identifies a clade containing these two isolates that sits outside the clades defining *R. mucilaginosa* and all other known *Rothia* species (Fig. 2a, b). We therefore conclude that these isolates represent a new species that we have named *Rothia gullae* (Table 2). Interestingly, we found a discrepancy between analyses based on ANI and phylogeny, in that the clade defining *Rothia gullae* also contains two of our MAGs recovered from a single but different UK patient, even though these sit outside the 95 % ANI radius for the species. Comparisons between the genomes of the cultured isolates and the MAGs showed that the cultured isolates contained two ~30 kb segments absent from the MAGs. blastn searches (data not shown) show that one of these segments is closely related to a putative extracellular polysaccharide locus in *R. mucilaginosa* strain DY-18 (residues 1 766 922 to 1 794 192 in GenBank assembly AP011540.1), while the other represents a prophage closely related to *Siphoviridae* sp. isolate ct6vJ12 (GenBank assembly BK035779.1). Mapping metagenomic reads to these segments showed that they were absent from the metagenomes that produced the relevant MAGs, suggesting that these segments represent genuine genome differences rather than deficiencies in binning.

**Table 2.** Protologues for newly named species. Protologues for new *species* identified by culture or by analysis of metagenome-assembled genomes from human oesophageal or saliva samples

Species	Grammar and etymology	Description
*Rothia gullae* sp. nov.	gul.lae. L. gen, fem. n. *gullae*, of the gullet	A bacterial species cultured from the human oesophagus and assigned to this genus according to the algorithms of the GTDB Toolkit operating on GTDB Release R207 [17, 54]. The type strain is P3C3.S176, which has been submitted for deposition in NCTC and DSMZ. This species includes all bacteria with genomes that show ≥95 % average nucleotide identity to the genome of the type strain, which is available via NCBI BioProject PRJNA838635. The GC content of the type strain is 58.97 % and the genome length is 2.18 Mbp. Further information can be found in the Methods and in Table S4.
*Candidatus* Granulicatella gullae sp. nov.	gul.lae. L. gen, fem. n. *gullae*, of the gullet	A bacterial species identified by metagenomic analysis of a sample from the human oesophagus and assigned to this genus according to the algorithms of the GTDB Toolkit operating on GTDB Release R207 [17, 54]. This species includes all bacteria with genomes that show≥95 % average nucleotide identity to the type genome for the species to which we have assigned the MAG ID P6S.S16.bin.50.1 and which is available via NCBI BioProject PRJNA838635. The GC content of the type genome is 40.42 % and the genome length is 1.59 Mbp. Further information can be found in the Methods and in Table S4.
*Candidatus* Streptococcus gullae sp. nov.	gul.lae.L. gen, fem. n. *gullae*, of the gullet	A bacterial species identified by metagenomic analysis of a sample from the human oesophagus and assigned to this genus according to the algorithms of the GTDB Toolkit operating on GTDB Release R207 [17, 54]. This species includes all bacteria with genomes that show≥95 % average nucleotide identity to the type genome for the species to which we have assigned the MAG ID ERR2373089.bin.001 and which is available via NCBI BioProject PRJNA838635. The GC content of the type genome is 40.06 % and the genome length is 2.09 Mbp. Further information can be found in the Methods and in Table S4.
*Candidatus* Haemophilus gullae. sp. nov.	gul.lae.L. gen, fem. n. *gullae*, of the gullet	A bacterial species identified by metagenomic analysis of a sample from the human oesophagus and assigned to this genus according to the algorithms of the GTDB Toolkit operating on GTDB Release R207 [17, 54]. This species includes all bacteria with genomes that show≥95 % average nucleotide identity to the type genome for the species to which we have assigned the MAG ID ERR2373136_bin.10 and which is available via NCBI BioProject PRJNA838635. The GC content of the type genome is 40.04 % and the genome length is 2.10 Mbp. Further information can be found in the Methods and in Table S4.
*Candidatus* Nanosynbacter quadrami. sp. nov.	quad.ra’mi. N.L. gen. n. *quadrami* of the Quadram Institute, where the species was discovered	A bacterial species identified by metagenomic analysis of a sample from the human oesophagus and assigned to this genus according to the algorithms of the GTDB Toolkit operating on GTDB Release R207 [17, 54]. This species includes all bacteria with genomes that show≥95 % average nucleotide identity to the type genome for the species to which we have assigned the MAG ID ERR2373117.bin.7 and which is available via NCBI BioProject PRJNA838635. The GC content of the type genome is 43.15 % and the genome length is 0.74 Mbp. Further information can be found in the Methods and in Table S4.
*Candidatus* Nanosynbacter gullae. sp. nov.	*gullae* L. gen, fem. n. gullae, of the gullet	A bacterial species identified by metagenomic analysis of a sample from the human oesophagus and assigned to this genus according to the algorithms of the GTDB Toolkit operating on GTDB Release R207 [17, 54]. This species includes all bacteria with genomes that show≥95 % average nucleotide identity to the type genome for the species to which we have assigned the MAG ID P11B.S7.bin.28.1 and which is available via NCBI BioProject PRJNA838635. The GC content of the type genome is 43.99 % and the genome length is 0.67 Mbp. Further information can be found in the Methods and in Table S4.
*Candidatus* Nanosynbacter colneyensis. sp. nov.	col.ney.en’sis. N.L. fem. adj. *colneyensis* pertaining to Colney, the Norfolk village which is home to the Quadram Institute where the species was first described	A bacterial species identified by metagenomic analysis of a sample from the human oesophagus and assigned to this genus according to the algorithms of the GTDB Toolkit operating on GTDB Release R207 [17, 54]. This species includes all bacteria with genomes that show≥95 % average nucleotide identity to the type genome for the species to which we have assigned the MAG ID P2B.S1.bin.0.1 and which is available via NCBI BioProject PRJNA838635. The GC content of the type genome is 43.65 % and the genome length is 0.66 Mbp. Further information can be found in the Methods and in Table S4.
*Candidatus* Nanosynbacter norwichensis. sp. nov.	nor.wich.en’sis. N.L. masc. adj. *norwichensis* pertaining to English city of Norwich, which is home to the Quadram Institute where the species was first described.	A bacterial species identified by metagenomic analysis of a sample from the human oesophagus and assigned to this genus according to the algorithms of the GTDB Toolkit operating on GTDB Release R207 [17, 54]. This species includes all bacteria with genomes that show≥95 % average nucleotide identity to the type genome for the species to which we have assigned the MAG ID P5B.S4.bin.39.1 and which is available via NCBI BioProject PRJNA838635. The GC content of the type genome is 43.52 % and the genome length is 0.74 Mbp. Further information can be found in the Methods and in Table S4.
*Candidatus* Nanosyncoccus oralis. sp. nov.	o.ra’lis. L. masc./fem. adj. *oralis,* of the mouth, the source of the first isolate.	A bacterial species identified by metagenomic analysis of a sample of human saliva and assigned to this genus according to the algorithms of the GTDB Toolkit operating on GTDB Release R207 [17, 54]. This species includes all bacteria with genomes that show≥95 % average nucleotide identity to the type genome for the species to which we have assigned the MAG ID P13S.S20.bin.18.1 and which is available via NCBI BioProject PRJNA838635. The GC content of the type genome is 42.53 % and the genome length is 0.57 Mbp. Further information can be found in the Methods and in Table S4.

### Metagenome-assembled genomes

After host-read depletion, >73 million reads were recovered from the eleven oesophageal metagenomes generated in this study, with an average of 6.7 million metagenomic reads per sample. More than 79 million host-depleted reads were recovered from the 41 oesophageal metagenomes from a recent Australian study [7], with an average of 1.9 million metagenomic reads per sample (Table S3).

**Fig. 3.**
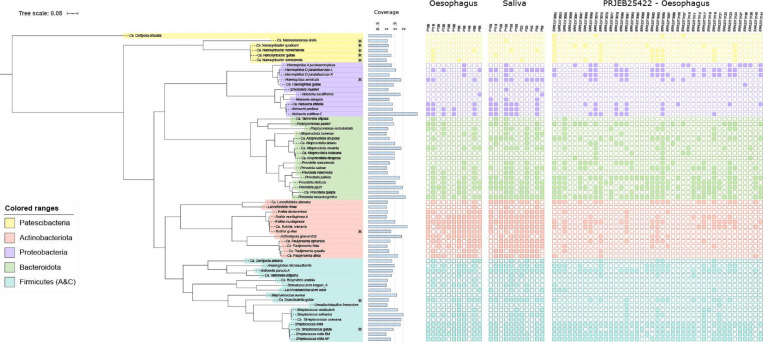
Phylogenetic tree of 63 bacterial species recovered from the oesophagus and saliva of 52 healthy human patients undergoing endoscopy. Oesophageal samples were recovered from 41 patients recruited in study PRJEB25422 and eleven patients recruited as part of this study (PRJNA838635). Saliva samples were recovered from the eleven patients recruited as part of this study. Phylum is indicated by colour range and star symbols indicate species novelty. All novel species alongside species assigned GTDB alphanumeric placeholder designations have been provided with new Latinate names. Species presence within described metagenomic samples is indicated by a filled square block, with presence determined at 1X mean genome coverage (proportion of nucleotides in a genome covered by at least one read) over at least 25 % of the genome length. The tree was reconstructed using PhyloPhlAN 3.0.58 against 400 marker genes before reconstruction using FastTree and RAxMLof a MAFFT sequence alignment. The resulting tree was visualised using the online iTOL v5.7 tool.

Assemblies from host-genome-depleted samples generated 722, 527 contigs longer than 1000 bp, which were assigned to 489 genomic bins. One hundred and thirty-six of these bins represent medium or high-quality MAGs with>10X coverage in their source metagenome (Table S4). Around two thirds of these MAGs (52 from saliva; 36 from the oesophagus) were derived from UK samples, while the remainder (*n*=48) were derived from the Australian samples from BioProject PRJEB25422, described by Deshpande *et al.* [7]. Clustering at 95 % ANI followed by analysis using the GTDB toolkit resulted in 56 species clusters, spanning 25 genera and seven of the bacterial phyla listed in GTDB; *Actinobacteriota, Bacteroidota, Patescibacteria, Proteobacteria, Firmicutes, Firmicutes_A* and *Firmicutes_C* (Fig. 3, Table S5, available in the online Supplementary Material). Thirty-seven of these species have cultured type strains, whereas 19 remain uncultured and represented only by MAGs. Most of these species and all of the genera have been reported from the oral cavity or upper respiratory tract, but for most this represents the first evidence of their occurrence in the oesophagus. Five of the twelve species recovered from oesophageal samples by bacterial culture were also recovered by metagenomic binning.

Eight of our metagenomic species clusters remain unclassified according to the GTDB toolkit and phylogenetic analysis confirms that these species clusters sit outside the clades defining known species within the same genus (Fig. S1, available in the online version of this article). We have therefore assigned these species novel *Candidatus* names: *Ca.* Granulicatella gullae, *Ca.* Streptococcus gullae, *Ca.* Nanosynbacter quadramensis, *Ca.* Nanosynbacter gullae, *Ca.* Nanosynbacter colneyensis, *Ca.* Nanosynbacter norwichensis, *Ca.* Nanosynococcus oralis and *Ca.* Haemophilus gullae. (Table 2) These novel species show 10 % relative abundance in the oesophageal microbiome and account for just over 5 % of the salivary microbiome.

Interestingly, five of these novel species (from the genera *Ca.* Nanosynbacter and *Ca.* Nanosynococcus) – along with one placeholder GTDB species *Ca.* Clofiposa ofocaria – belong to the recently described phylum *Patescibacteria* (largely synonymous with the CPR). Consistent with the view that such bacteria live as epibionts, all six MAGs assigned to this phylum showed small genome sizes (<900 kb). Although *Patescibacteria* are known to inhabit the oral cavity, this is the first report of their presence in the oesophagus.

### Species catalogue

Our non-redundant species catalogue contains 63 species derived from cultured isolates or from recovered MAGs. Mapping revealed that these species account for around half of the sequences in the oesophageal and saliva metagenomes. Nineteen of these species are currently identified solely by user-unfriendly alphanumeric placeholder designations in GTDB. Use of the Latinate species names recently published by Pallen *et al.* [32] has provided us with short practical alternatives (Table 1).

**Fig. 4.**
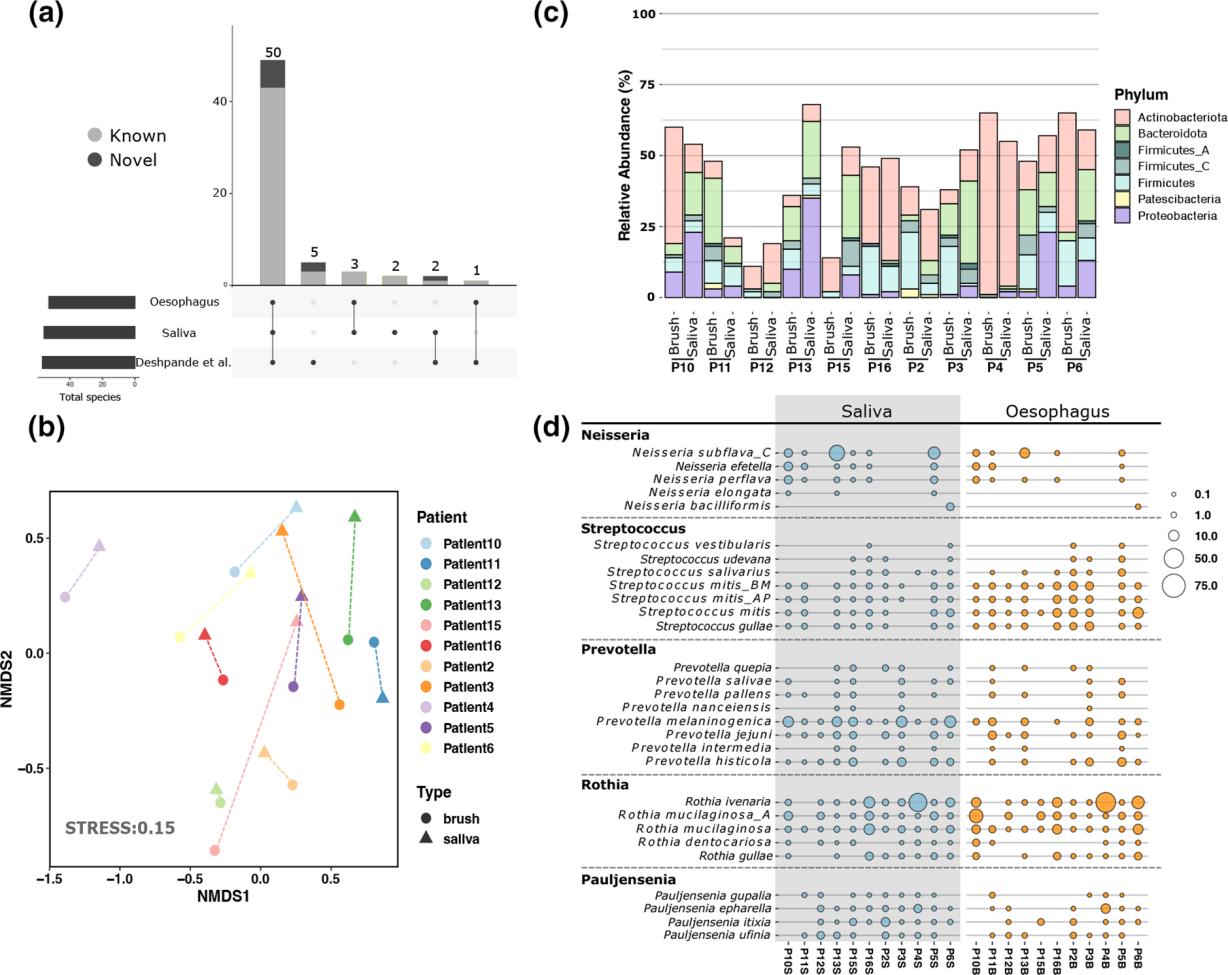
Distribution and abundance of bacterial species recovered from the healthy human oesophagus and saliva across metagenomic samples. (**a**) Upset plot depicting presence of 63 metagenomic species across metagenomic samples from BioProjects PRJEB25422 and PRJNA838635. Samples derived from PRJNA838635 have been further categorised as being either oesophageal or salivary metagenomes. Bar colour indicates species novelty. (**b**) Nonmetric multidimensional scaling (NMDS) of Bray-Curtis dissimilarity for 63 recovered bacterial species within the oesophagus and saliva of eleven healthy patients. Dissimilarity matrix was based upon normalised relative abundance of species across metagenomes of PRJNA838635. Analysis of similarities (ANOSIM) was used for statistical testing of similarity (R=0.82, *P*=0.02). Colour depicts source patient while shape depicts sample type. (**c**). Normalised relative abundance (percent) of phyla within oesophageal and salivary metagenomes of BioProject PRJNA838635. Samples are shown for eleven patients. (**d**). Bubble plot showing the normalised relative abundance (percent) of species from the five predominant genera (*Neisseria, Pauljensenia, Prevotella, Rothia and Streptococcus*) within oesophageal and saliva samples of BioProject PRJNA838635. Relative abundance is indicated by bubble size while bubble colour depicts sample source.

No species was present in all oesophageal samples. Mapping also showed that 60 species occurred in at least one oesophageal metagenome from either study, with the majority (*n*=50) identified in both cohorts (Fig. 4a). Although we cultured *Staphylococcus aureus* from two patients, this organism was not identified within any of the oesophageal or salivary metagenomes. We observed significant clustering of samples according to individual (R=0.6, *P*=0.0001; Fig. 4b), but not according to sample type (saliva versus oesophagus), suggesting that the oesophageal microbiome is closely related to the salivary microbiome within an individual. Within-species MAGs recovered from the oesophagus and saliva from the same individual showed higher similarity than that seen between MAGs of the same species recovered from different people. The oesophageal microbiome of our eleven patients was dominated by three genera (* Streptococcus *, *Rothia* and *Prevotella *), with the addition of two further genera in the saliva (* Pauljensenia* and *Neisseria *) (Fig. 4c). The presence and abundance of species from these genera varied considerably within the saliva and oesophagus of individual patients, with the same genera predominating at both sites only identified in two patients (Fig. 4d).

## Discussion

Compared to the lower gut, the microbiology of the human oesophagus remains largely unexplored. Here, in recovering over a hundred bacterial genomes through culture and metagenomic analysis, we have obtained the first high-resolution view of microbial diversity within this important environment. Although contamination with host DNA presents a potential challenge when analysing metagenomic samples, here we have shown that it is possible to retrieve enough sequence data to enable recovery of MAGs from oesophageal brushings.

Remarkably, from this everyday setting, we have discovered one new cultured species and eight novel *Candidatus* species, paving the way for detailed characterisation of these newfound taxa, including culture of the *Candidatus* species. Not only have we discovered new species within well-characterised genera, such as *Strepotococcus* and *Haemophilus *, but we have also found six species from the enigmatic *Patescibacteria *, which are thought to live as epibionts in close association with other bacteria in this environment [46]. Identification of the partners of these epibionts presents an interesting challenge for the future.

The fact that no one species was found in all oesophageal samples suggests that, as with the lower gut, there is no core human oesophageal microbiome. Similarly, evidence of clustering by person rather than by sample suggests that the oesophageal microbiome is closely related to the oral microbiome within the same individual. We found no evidence in our sample sets of the bacterial species proposed to play a role in progression toward cancer, *Campylobacter concisus* [47] and *Fusobacterium nucleatum* [48]. Now established in metagenomic recovery of genomes from the oesophagus, the techniques described here can be used in future studies associated with oesophageal pathologies.

## Conclusions

Recovery of genomes and discovery of new species represents an important step forward in our understanding of the oesophageal microbiome. The genes and genomes that we have released into the public domain, along with the methodologies we have pioneered in this preliminary study, will provide a base line for future more definitive catalogues, plus comparative, mechanistic and intervention studies.

## Supplementary Data

Supplementary material 1

Supplementary material 2
