# Supplementary material for: An initial genomic blueprint of the healthy human oesophageal microbiome
Source: Access Microbiol. 2023 Jun 26;5(6):acmi000558.v3. doi: 10.1099/acmi.0.000558.v3 (PMC10323806; doi:10.1099/acmi.0.000558.v3)

Figure S1: Phylogenetic placement of novel species

Phylogenetic placement of MAG P6S.S16.bin.50.1, which we have assigned to a new species named *Ca.* Granulicatella gullae

Phylogenetic placement of MAGs ERR2373117_bin.7**,** P11B_S7_bin.2.28, P2B_S1_bin.0.1 and P5B_S4_bin.39.1.1, which we have assigned to the new specie**s** *Ca.* Nanosynbacter quadrami *Ca.* Nanosynbacter gullae, *Ca.* Nanosynbacter colneyensis, *Ca.* Nanosynbacter norwichensis respectively.

Phylogenetic placement of MAG P13S_S20_bin.18.1, which we have assigned to the new species *Ca.* Nanosyncoccus oralis.

Phylogenetic placement of MAG ERR2373089.bin.001, which we have assigned to the new species *Ca.* Streptococcus gullae.

Phylogenetic placement of MAG ERR2373136 concoct.10 which we have assigned to the new species *Ca.* Haemophilus gullae

Phylogenetic placement of MAG P6S.S16.bin.50.1, which we have assigned to a new species named *Ca.* Granulicatella gullae

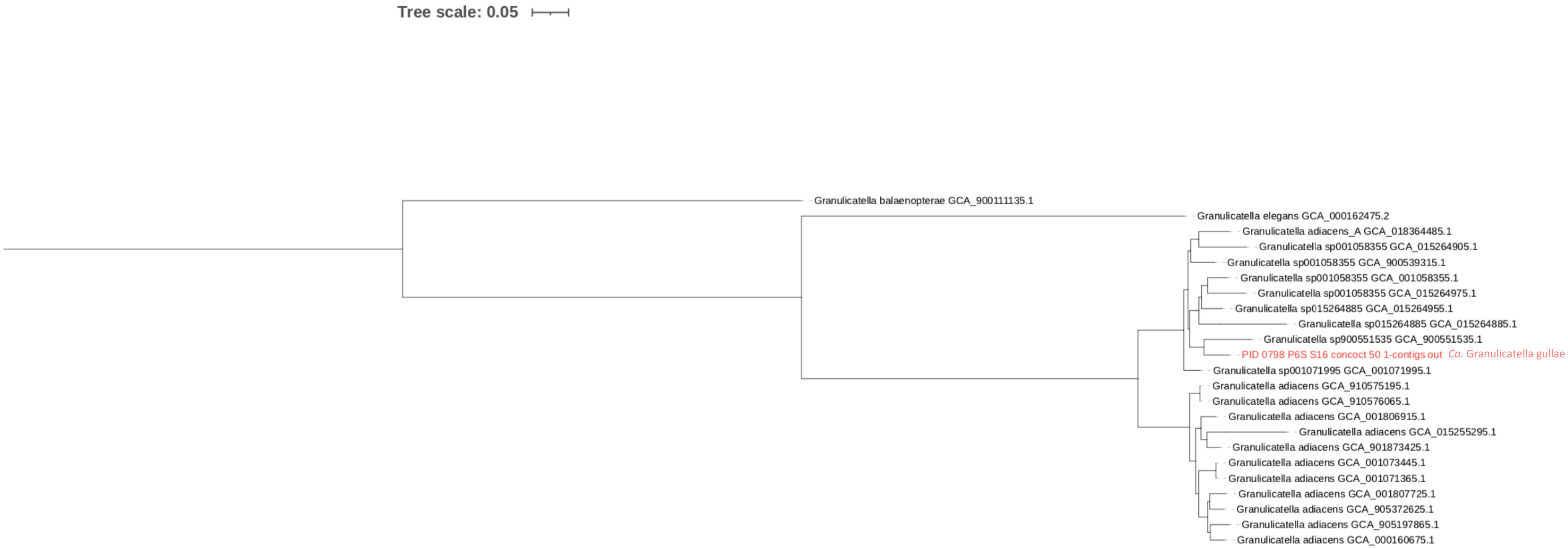

Phylogenetic placement of MAGs ERR2373117_bin.7**,** P11B_S7_bin.2.28, P2B_S1_bin.0.1 and P5B_S4_bin.39.1.1, which we have assigned to the new specie**s** *Ca.* Nanosynbacter quadrami, *Ca.* Nanosynbacter gullae, *Ca.* Nanosynbacter colneyensis, *Ca.* Nanosynbacter norwichensis respectively.

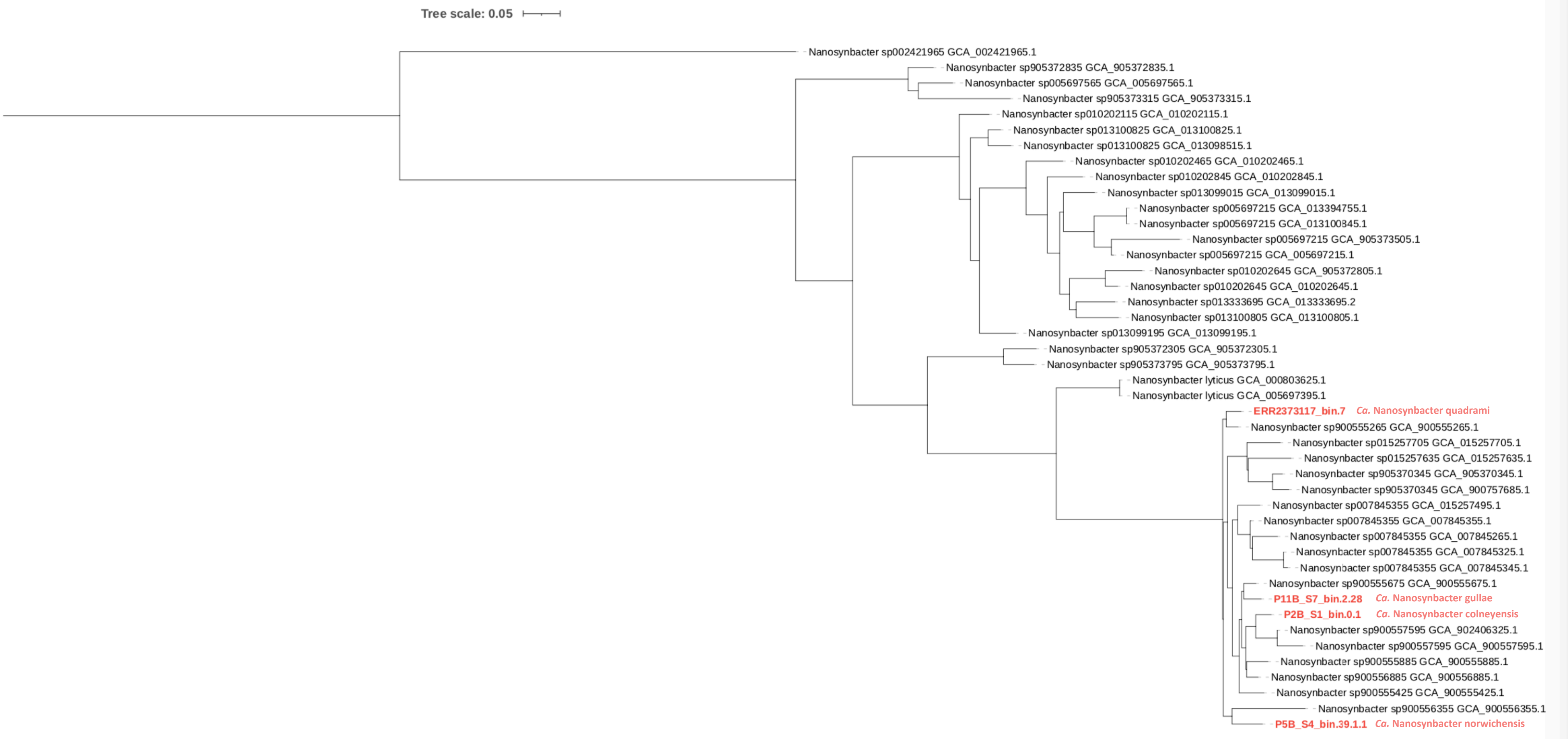

Phylogenetic placement of MAG P13S_S20_bin.18.1, which we have assigned to the new species Ca. Nanosyncoccus oralis.

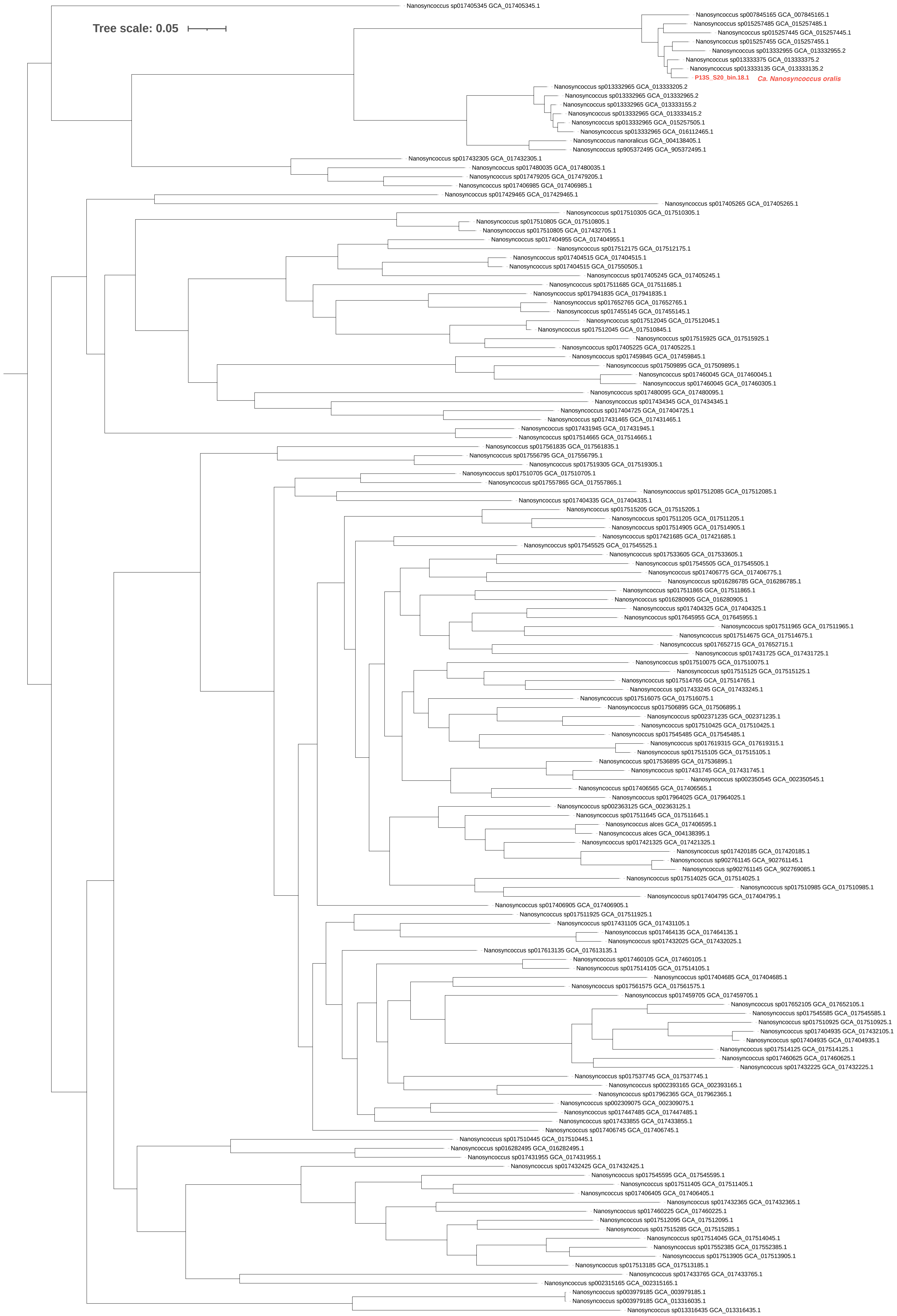

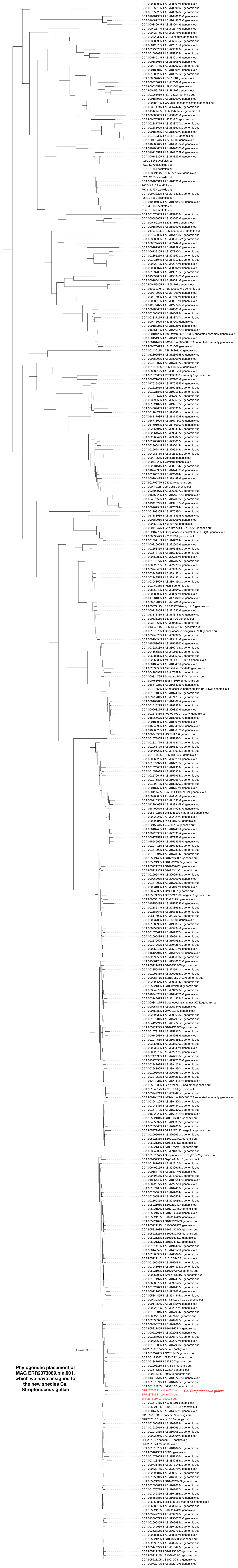

Phylogenetic placement of MAG ERR2373089.bin.001, which we have assigned to the new species *Ca.* Streptococcus gulliae

Tree scale: 0.1

Phylogenetic placement of MAG ERR2373136 concoct.10 which we have assigned to the new species Ca. Haemophilus gullae

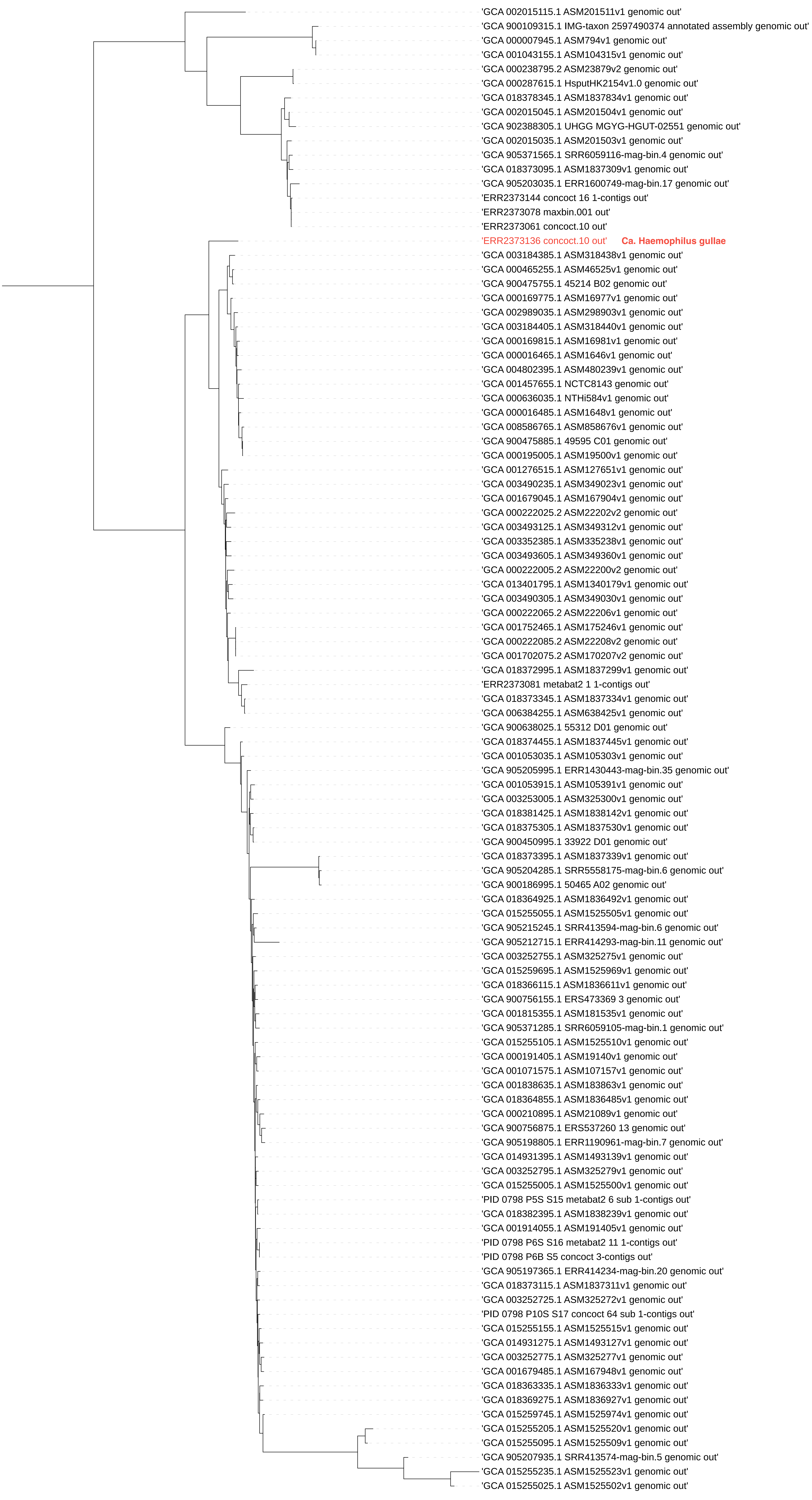

Supplement: Supplementary material 2 [file acmi-5-558.v3-s001.pdf]
